# Extraocularly Manipulated Flangeless Intrascleral Haptic Fixation of Intraocular Lens Using Zigzag Needles

**DOI:** 10.7759/cureus.116508

**Published:** 2026-09-18

**Authors:** Ryo Sakata, Masayuki Akimoto

**Affiliations:** 1 Ophthalmology, Osaka Red Cross Hospital, Osaka, JPN

**Keywords:** aphakia, flangeless, intrascleral intraocular lens fixation, secondary iol implantation, yamane technique

## Abstract

Purpose: To evaluate a novel, flangeless intrascleral intraocular lens (IOL) fixation technique using zigzag 27-gauge needles that prioritizes extraocular maneuvers and eliminates the need for sutures, heat-based flanging, or specialized equipment.

Methods: This study evaluated patients who underwent intrascleral IOL fixation with 27-gauge needles at Osaka Red Cross Hospital between March and September 2024. The surgical procedure was performed using a three-piece NX-70 IOL. Standard 27-gauge needles were bent (the leading needle was bent at a slightly obtuse angle and the trailing needle was bent in a zigzag shape in three stages) to guide the IOL haptics extraocularly. Custom silicone pieces (approximately 1 mm, cut from a standard phaco-sleeve) were temporarily attached to the haptic ends to secure them during the process. The haptics were then retracted into limbal-parallel scleral tunnels without flange fixation. Patients were evaluated preoperatively and at one month postoperatively for best-corrected visual acuity (BCVA), corneal endothelial cell density (ECD), and IOL tilt.

Results: Eighteen eyes of 17 patients (mean age: 74.3 ± 11.4 years) were included. At one month postoperatively, the mean BCVA improved from 0.56 ± 0.66 logMAR to 0.15 ± 0.40 logMAR. The mean ECD decreased from 2243 ± 319 cells/mm² to 1999 ± 502 cells/mm². The mean IOL tilt was 5.74 ± 2.95 degrees. Postoperative complications occurred in one eye each (5.6%): vitreous hemorrhage (which resolved within two weeks), temporary intraocular pressure elevation, transient corneal edema, and one intraoperative rhegmatogenous retinal detachment (unrelated to the IOL fixation technique itself). IOL repositioning due to tilt was required in one patient (5.6%), and no cases of iris capture were observed.

Conclusions: This modified flangeless intrascleral IOL fixation technique using zigzag-bent 27-gauge needles was feasible using standard surgical instruments and yielded acceptable preliminary short-term clinical outcomes in this small case series. Larger comparative studies with longer follow-up are required to evaluate long-term IOL stability and safety.

## Introduction

In ophthalmic surgery, implantation of an intraocular lens (IOL) in the absence of adequate capsular support has been a significant challenge. Intrascleral IOL fixation techniques have gained popularity in recent years. In 2007, Gabor and Agarwal first introduced an intrascleral IOL fixation technique, eliminating the need for sutures while reducing the risks associated with them [1, 2]. Further advancements have led to the development of the double-needle-flanged technique by Yamane et al., which utilizes 30-gauge thin-wall needles to externalize the IOL haptics and create a flange at the end of each haptic for secure fixation [3]. This technique has gained popularity because it simplifies the traditional two-step process of externalizing the IOL haptics and inserting them into scleral tunnels into a single-step procedure. However, intraocular manipulation and precise lens positioning remain challenging. Owing to these factors, IOL dislocation or exposure of the flanged haptic has been reported, and IOL repositioning can occasionally be challenging. Techniques utilizing a microtube for external manipulation of IOL haptics have also been reported [4-6]. While these methods effectively reduce the need for intraocular maneuvers, they require specialized microtube equipment.

Therefore, we developed a surgical technique that prioritizes extraocular maneuvers [7, 8]. Similarly, several techniques for performing modified double-needle-flanged extraocular fixation have been reported [9-13]. However, to the best of our knowledge, no existing method has enabled the implantation of a three-piece acrylic IOL into a scleral tunnel without flange fixation in a single-step procedure. Herein, we describe this modified flangeless technique and evaluate its feasibility and preliminary short-term clinical outcomes in patients undergoing secondary intrascleral IOL fixation.

The data were presented at the 78th Annual Congress of the Japan Clinical Ophthalmology, Kyoto, Japan, in November 2024, and at the annual meeting of the American Society of Cataract and Refractive Surgery (ASCRS), Los Angeles, California, in May 2025.

## Materials and methods

Study participants

During the study period, 21 eyes of 20 patients underwent the described intrascleral IOL fixation procedure with 27-gauge needles (Nipro, Osaka, Japan) at Osaka Red Cross Hospital between March and September 2024. Flange formation was additionally performed in three eyes. Because the purpose of the present study was to evaluate the flangeless fixation technique, these three eyes were excluded from the analysis. Therefore, the final study cohort consisted of 18 eyes of 17 patients treated exclusively with flangeless fixation. All procedures were performed by the same surgeon (M.A.). The study protocol was approved by the hospital’s Institutional Review Committee, and all procedures were performed in accordance with the Declaration of Helsinki. Informed consent was obtained from each patient prior to surgery. The inclusion criteria were aphakia, dislocated IOL, subluxated crystalline lens, and consent to participate. The exclusion criteria included retinal diseases requiring treatment, such as retinal detachment or macular pucker, preoperative intraocular pressure (IOP) ≥ 25 mmHg despite treatment with eye drops, scleritis, age < 20 years, postoperative follow-up of less than one month, and flange formation during the procedure.

Outcome measures

Preoperative and postoperative evaluations included best-corrected visual acuity (BCVA), slit-lamp inspection, IOP assessment, and dilated indirect slit-lamp biomicroscopy. Corneal endothelial cell density (ECD) was measured using specular microscopy before surgery and one month after surgery. IOL tilt was quantified using swept-source optical coherence tomography (SS-1000 CASIA-2; Tomey Co., Nagoya, Japan) one month after surgery.

IOL specification

This study used a three-piece IOL (NX-70 [Santen, Osaka, Japan]) coupled with an injector.

Surgical procedure

A short version of this surgical technique is shown in Supplementary Video 1.

**Video 1:** A shorter version of the technique. This video demonstrates a shorter version of the original technique.

The surgical procedure began with a conjunctival incision, followed by a 3.0-mm corneoscleral incision at the 11-12 o’clock meridian using a 3.0-mm slit knife (Mani, Tochigi, Japan). We performed complete pars plana vitrectomy before IOL fixation. To allow safe scleral needle passage, the globe was softened by venting through paracentesis and lowering infusion pressure to about 5 mmHg. The nasal and temporal sides of the sclera were marked approximately 90° from the main incision and 180° apart (Fig. 1A). Additional markings were placed 2 mm apart in a counterclockwise direction, with partial-thickness incisions made at each of the four marked points (Fig. 1B). A 7-mm three-piece IOL was loaded into the injector with the leading haptic slightly extended. Two fine incisions were made in the ultrasound sleeve, allowing for the insertion of two 27-gauge needles. Small silicone pieces were attached to the needles to temporarily secure the haptics (Fig. 1C). These silicone pieces were custom-cut (about 1 mm) from a phaco-sleeve packed in standard boxes (Alcon, Fort Worth, Texas) and were not proprietary devices. For the leading haptic, the guiding needle was bent at a slightly obtuse angle 6 mm from the tip and positioned below the main incision (Fig. 1D). The guiding needle for the trailing haptic was bent in three stages: 5 mm from the 9 o’clock limbus, 3 mm posteriorly, and finally bent upward (Fig. 1E). This zigzag needle was designed to allow the needle tip to emerge from the main incision and pass naturally through the pupil, scleral incision, and scleral tunnel. A third needle was bent at a right angle with a 3-mm tip (Fig. 1F). The scleral tunnel was created using the guiding needle for the leading haptic through partial-thickness incisions 2 mm behind the limbus, with the needle tip slightly exposed (Fig. 1G). The length of the limbal-parallel scleral tunnel was standardized at 2 mm in all cases. A second needle punctured the sclera, and after softening the globe to approximately 5 mmHg by venting the paracentesis and the lowering infusion pressure, the guiding needle was inserted into the eye and guided through the main incision using a retrieval needle (Fig. 1H). The leading haptic, which was exposed from the injector, was inserted into the guide needle. Once the haptic was firmly placed, the IOL was inserted (Fig. 1I).

The IOP was restored, and a third needle was inserted 2 mm behind the marked limbus, creating a scleral tunnel. A zigzag-bent guiding needle for the trailing haptic was carefully exchanged for and inserted into the tunnel to avoid damaging it (Fig. 1J). This procedure was repeated for the trailing haptic, which was guided through the main incision using a retrieval needle (Fig. 1K). The trailing haptic was inserted into a zigzag-bent guiding needle using a needle holder (Fig. 1L). To prevent unplanned retraction due to IOP fluctuations during the procedure, small silicone pieces were temporarily attached to the tips of both the leading and trailing haptics (Fig. 1M).

Subsequently, the silicone sleeves were removed, and the haptics were retracted into the scleral tunnels as intended (Fig. 1N). Peripheral iridotomy was performed using a vitrectomy cutter, and IOL alignment was confirmed (Fig. 1O).

**Figure 1:**
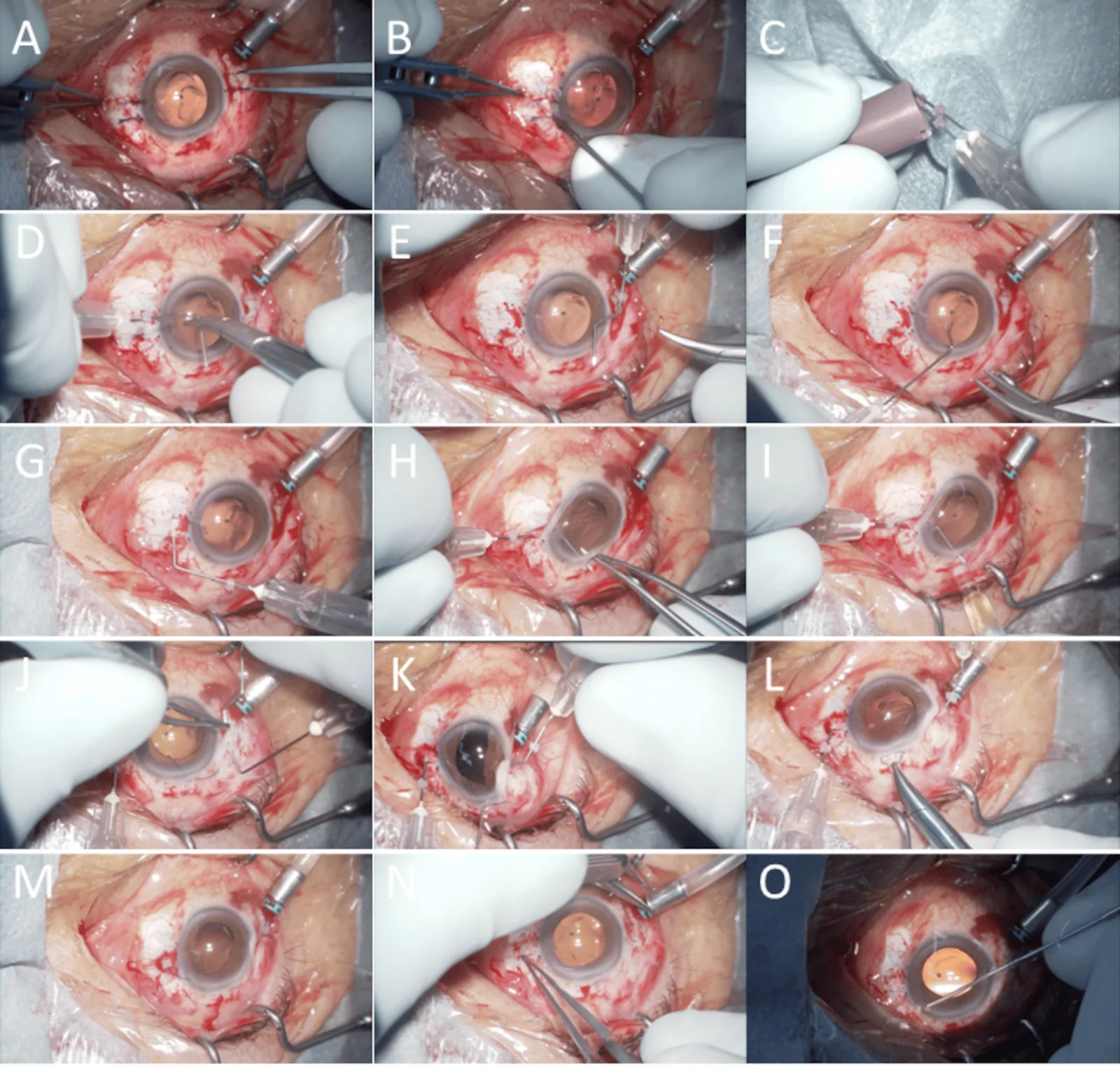
Sequential illustration of the surgical technique for intrascleral intraocular lens (IOL) fixation. (A) Marking of the nasal and temporal sides. (B) Creation of radial scleral grooves. (C) Creation of small silicone pieces cut from a phaco-sleeve. (D-F) Bending of 27G needles (Nipro, Osaka, Japan). (G) Inserting the guiding needle into the scleral tunnel. (H, I) Extruding haptics extraocularly. (J) Inserting the zigzag-bent guiding needle into the scleral tunnel. (K, L) Extruding haptics extraocularly. (M) Affixation of a silicone piece to the trailing haptic to prevent retraction. (N) Final placement of both haptics. (O) Peripheral iridotomy. IOL: intraocular lens.

Statistical analysis

Continuous variables are presented as mean ± standard deviation, and categorical variables are presented as numbers and percentages. Preoperative and one-month postoperative BCVA and corneal ECD were compared using the Wilcoxon signed-rank test. Paired observations with a difference of zero were excluded from the analysis. Because of the small sample size, statistical significance was determined by comparing the Wilcoxon signed-rank test statistic (T), defined as the smaller of the sums of the positive and negative ranks, with the corresponding critical value for a two-sided significance level of 0.05. Statistical calculations were performed using Microsoft Excel, Version 2021 (Microsoft Corporation; Redmond, Washington). A two-sided significance level of 0.05 was used.

## Results

Patient characteristics are shown in Table 1. Eighteen eyes, including 11 IOL-dislocated eyes and 7 lens-dislocated eyes, of 17 patients were treated using this new technique. Among them, 15 eyes (83.3%) were from male patients, and 3 eyes (16.7%) were from female patients. The mean patient age at the time of surgery was 74.3 ± 11.4 (range: 55-89) years. The mean axial length was 25.34 ± 1.82 mm. The mean BCVA was 0.56 ± 0.66 logMAR, which improved to 0.15 ± 0.40 logMAR at one month. This improvement was statistically significant according to the Wilcoxon signed-rank test (T = 13, n = 16, critical value = 29; two-sided α = 0.05). The mean corneal ECD decreased from 2243 ± 319 cells/mm^2^ to 1992 ± 502 cells/mm^2^ at one month. This decrease was also statistically significant according to the Wilcoxon signed-rank test (T = 35.5, n = 18, critical value = 40; two-sided α = 0.05). The mean IOL tilt at one month was 5.74 ± 2.95°. In the eye that required IOL repositioning, the one-month tilt measurement was obtained after repositioning. The mean refractive difference from the predicted value calculated using the Sanders-Retzlaff-Kraff trial formula or Barrett True AL formula for in-the-bag fixation was -0.26 ± 0.84 diopters (D).

**Table 1:** Patient characteristics and data. Data are presented as means ± standard deviations where applicable. AL: axial length; IOP: intraocular pressure; IOL: intraocular lens; logMAR BCVA: logarithm of the minimal angle of resolution best-corrected visual acuity; D: diopter; ECD: endothelial cell density.

Variable	Value
Eyes, n	18
Age, years (range)	74.3 ± 11.4 (55-89)
Sex: Male, n (%)	15 (83.3)
Sex: Female, n (%)	3 (16.7)
Diagnosis: Aphakia, n (%)	0 (0)
Diagnosis: IOL dislocation, n (%)	11 (61.1)
Diagnosis: Lens dislocation, n (%)	7 (38.9)
AL, mm	25.34 ± 1.82
Refractive errors (spherical equivalent), diopters (D)	0.18 ± 4.73
IOP, mmHg	17.5 ± 4.0
Baseline logMAR BCVA	0.56 ± 0.66
logMAR BCVA at one month	0.15 ± 0.40
Baseline corneal ECD (cells/mm^2^)	2243 ± 319
Corneal ECD at one month (cells/mm^2^)	1992 ± 502
IOL model	NX-70 (Santen)
IOL power, diopters (D)	18.3 ± 3.7
Refractive difference, diopters (D)	-0.26 ± 0.84
IOL tilt (^o^)	5.74 ± 2.95

Postoperative complications included vitreous hemorrhage in one eye (5.6%), temporary IOP elevation in one eye (5.6%), transient corneal edema in one eye (5.6%), and intraoperative rhegmatogenous retinal detachment in one eye (5.6%; Table 2). The vitreous hemorrhage disappeared within two weeks of surgery. Iris capture of the IOL was not observed in any patient, and IOL repositioning for IOL tilt was performed in one patient (5.6%).

**Table 2:** Postoperative complications. IOP: intraocular pressure; IOL: intraocular lens.

Variable	Value
Eyes, n	18
Temporary corneal edema, n (%)	1 (5.6)
Vitreous hemorrhage, n (%)	1 (5.6)
Rhegmatogenous retinal detachment, n (%)	1 (5.6)
Temporary hypotony, n (%)	0 (0)
Temporary IOP elevation, n (%)	1 (5.6)
Iris capture, n (%)	0 (0)
Repositioning of the IOL, n (%)	1 (5.6)
Cystoid macular edema, n (%)	0 (0)

## Discussion

The double-needle-flanged technique utilizing 30G thin-wall needles is generally considered simple and minimally invasive, even without the need for a conjunctival incision [3]. However, certain challenges remain, particularly with regard to intraocular manipulation, angled sclerotomy, and precise lens-positioning adjustment. For beginners, inserting the IOL haptic into the needle can be particularly difficult because of the narrow intraocular space, and maintaining a consistent shortening of the haptic while forming a flange is technically demanding. Therefore, many surgeons have sought to modify and improve the technique to enhance its reliability and ease of use [8-13]. In our study, we aimed to develop a more user-friendly method for intrascleral fixation of the IOL, with a focus on reducing the need for complex intraocular manipulations and specialized equipment. Referring to the double-needle flanged technique, we altered each step to design a procedure that used commonly available instruments and incorporated elements from previously reported techniques.

Observed perioperative complications in the present series included vitreous hemorrhage, transient IOP elevation, transient corneal edema, and intraoperative rhegmatogenous retinal detachment. Previous studies of scleral IOL fixation have reported a range of complications, including vitreous hemorrhage and transient hypotony, as summarized in Table 3 [3, 8, 14, 15]. However, these values should not be interpreted as direct comparisons because the studies differed in patient populations, surgical techniques, sample sizes, follow-up durations, and definitions of complications. Therefore, these historical data are provided only as descriptive context.

**Table 3:** Comparison of surgical results with other techniques of intrascleral fixation of intraocular lens. Data are presented as means ± standard deviations where applicable. IOL: Intraocular lens; ECD: endothelial cell density.

Surgical results	Our study	Yamane et al. [3]	Morino et al. [8]	Kelkar et al. [14]	Ishikawa et al. [15]
Eyes, n	18	100	20	31	29
Follow-up (month)	1	20.6 ± 10.0	3	6	3
IOL model	NX-70	NX-70	NX-70	Not available	NX-70
Refractive difference, diopters (D)	-0.26 ± 0.84	-0.41 ± 0.98	-0.65 ± 0.34	-0.75	0.46 ± 0.84
IOL tilt (°)	5.74 ± 2.95	3.83 ± 2.69	7.5 ± 3.8	Not available	Not available
ECD loss (%)	-10.87 ± 18.76	Not available	-11.0 ± 13.8	Not available	-17.2 ± 18.3

Repositioning for IOL tilt was required in one eye (5.6%). Although the intended sclerotomy location was 2 mm posterior to the limbus, in this eye, the sclerotomy was inadvertently created more than 2 mm posterior to the limbus because of a technical error. Inaccurate or asymmetric sclerotomy positioning may alter the effective fixation geometry of the haptics and contribute to postoperative IOL decentration or tilt. Intraoperative rhegmatogenous retinal detachment was observed in one patient (5.6%) treated with our method. Detachment occurred when posterior vitreous detachment was induced during vitrectomy before IOL fixation. Therefore, we considered this to be unrelated to our method.

In this study, no haptic slippage or extrusion was observed. Although the Yamane technique utilizing flange fixation has become a popular approach for intrascleral fixation, numerous studies have reported intrascleral fixation without flanging. We have performed intrascleral fixation of IOLs using a scleral tunnel created with a thin-walled 30-gauge needle using our former techniques [7, 8]; postoperative haptic slippage is extremely rare, provided that a tunnel insertion length of approximately 2 mm is secured. In our new technique, the haptic is retained within a standardized 2-mm limbal-parallel scleral tunnel. On the other hand, in young patients, such as those with atopic dermatitis, we have experienced cases of slippage, presumably because they tend to rub their eyes. Since young patients require long-term stability over decades, we added a flange after taking their background into consideration. Meanwhile, we have no experience with external protrusion when using the scleral tunnel technique. We believe this is because the tunnel is created parallel to the scleral surface. These factors were taken into consideration when designing the structural concept of this technique.

However, our preliminary observation was limited up to one month after the surgery, and the long-term stability of this flangeless fixation mechanism remains to be established. 

One of the principal advantages of our technique is its accessibility. The recent development of microtube-assisted scleral fixation has introduced additional methods for external IOL haptic insertion with limited intraocular maneuvers [4-6]. However, their reliance on specialized tubing restricts their widespread application. Unlike microtube-assisted methods, our technique can be performed using standard surgical tools. Our technique circumvents this limitation by promoting reproducibility across various clinical settings.

On the other hand, while intraocular manipulation is reduced, procedures involving multiple scleral punctures or the use of curved needles require technical precision, which may increase the complexity of the surgery. However, this technique is made possible by the flexible polyvinylidene fluoride (PVDF) haptics of the NX-70 IOL and may not be suitable for IOLs with stiffer polymethyl methacrylate (PMMA) haptics. At the same time, the absence of iris capture in our series may also be attributable to the large optic diameter (7 mm) of the NX-70 IOL.

Although promising, the new technique has certain limitations, including a short follow-up period and a single-arm study design. Because fixation is flangeless, long-term stability remains uncertain, and risks such as late haptic migration or tunnel widening cannot be excluded. In addition, the technique requires accurate needle bending, haptic manipulation, and symmetric sclerotomy placement, and its reproducibility may therefore be influenced by surgeon experience. Furthermore, because this technique was performed using an IOL with flexible PVDF haptics, its applicability to IOLs with different haptic materials or mechanical properties remains uncertain. Further studies with extended follow-up periods are required to confirm these preliminary findings and determine the long-term viability of our technique.

## Conclusions

We report a modified method that allows one-step haptic insertion into a scleral tunnel while prioritizing extraocular manipulation. Our technique provides a feasible and accessible approach for scleral fixation of IOLs without the need for heat-based flange formation or specialized equipment. Moreover, our method relies on an extraocular manipulation approach. However, given the small sample size, single-arm design, and one-month follow-up period, these findings should be considered preliminary. Larger comparative studies with longer follow-up periods are required to evaluate long-term IOL stability, haptic-related complications, and the relative advantages and limitations of this technique compared with established fixation methods.
